# ^99m^Tc-Labeled LyP-1 for SPECT Imaging of Triple Negative Breast Cancer

**DOI:** 10.1155/2019/9502712

**Published:** 2019-09-25

**Authors:** Ningning Song, Lingzhou Zhao, Meilin Zhu, Jinhua Zhao

**Affiliations:** ^1^Department of Nuclear Medicine, Shanghai General Hospital, Shanghai Jiao Tong University School of Medicine, Shanghai 200080, China; ^2^School of Basic Medical Sciences, Ningxia Medical University, Yinchuan 750004, Ningxia, China

## Abstract

Triple negative breast cancer (TNBC), the most aggressive breast cancer type, is associated with high mortality and recurrence rates. An active-targeted strategy based on homing peptides is an effective approach to diagnose and treat cancer as it can deliver imaging agents or therapeutic drugs into desired tissues and accumulate less into off-target tissues. As a homing peptide, LyP-1 has shown properties of targeting, internalization, and proapoptosis to TNBC. In the study, we designed a Technetium-99m- (^99m^Tc-) labeled LyP-1 and investigated its feasibility for targeted single-positron emission computed tomography (SPECT) imaging of TNBC. The results showed that the LyP-1 peptide had acceptable biocompatibility in the studied concentration range and could specifically bind to TNBC cells in vitro. ^99m^Tc-labeled LyP-1 showed high radiochemical purity and stability and could be used as a probe for targeted SPECT imaging of TNBC cells in vitro and in a TNBC tumor-bearing mouse model. Our findings indicate that this active-targeted strategy has great potential to be developed into a new imaging tool for TNBC diagnosis.

## 1. Introduction

Triple negative breast cancer (TNBC), accounting for approximately 20% of all breast cancer cases, is the most aggressive form of the disease [1] and is more likely to affect women aged >40 years, African Americans, and those with *BRCA1* mutation [2]. Most TNBC cases are at a more advanced stage at diagnosis [3]. Because TNBC does not express the estrogen receptor (ER), progesterone receptor (PR), and human epidermal growth factor receptor 2 (HER2), it does not effectively respond to endocrine therapy and anti-HER2-targeted therapy [4]. Moreover, there are no available appropriate targeted therapies, and cytotoxic chemotherapy remains the primary systemic treatment [3–5]. As a result of these aggressive characteristics, TNBC has higher rates of recurrence and death than non-TNBC subtypes of breast cancer [5]. Exploring novel and specific molecular to improve the diagnosis and treatment efficiency of TNBC remains a major challenge to scientists and clinicians.

An active-targeted system based on homing peptides is a promising method to deliver payloads, such as imaging agents or drugs into diseased tissue, and to avoid accumulation in healthy tissue [6–8]. Among the existing homing peptides, LyP-1 was identified by the phage display technology and can specifically recognize tumor lymphatics, tumor cells, and tumor-associated macrophages/myeloid cells [9]. LyP-1 is a cyclic nonapeptide with a sequence of CGNKRTRGC, the two cysteines forming a disulfide bond [10]. The potential receptor of LyP-1 is a mitochondrial matrix protein called p32, which is overexpressed and aberrantly localized at the cell surface [11] and in the nucleus [12] of certain tumors [13], such as MDA-MB-231 and 4T1 TNBC cell lines [14, 15]. LyP-1 also belongs to a class of tumor-penetrating peptides with the potential capacities of transvascular transport, cell penetration, and parenchymal penetration. The potential mechanism is through the neuropilin-1-dependent C-terminal C-end rule (CendR) pathway [16, 17]. In addition, the proapoptotic effect of LyP-1 on its targeted pathological cells makes it a unique tumor homing peptide [18, 19]. These properties prompted us to develop LyP-1-based imaging agents for TNBC.

Single-photon emission computed tomography(SPECT) is one of the most widely used imaging tools for quantifying radionuclide distribution in clinical use [20]. Technetium-99m (^99m^Tc) is the most commonly used SPECT radionuclide and covers almost 85% of all diagnostic nuclear medicine applications [21]. This isotope is ideal for diagnostic nuclear imaging due to intrinsic advantages such as appropriate half-life (6.02 h), low-energy *γ*-ray (140 KeV) and low production cost [22]. LyP-1 has been labeled with iodine-131 and is successfully being used to image mice-bearing MDA-MB-435 tumors [23]. However, there is a lack of studies focused on exploring the utility of ^99m^Tc-labeled LyP-1 in TNBC diagnosis and therapy.

In this study, we selected LyP-1 as a targeted ligand and ARAL (ARALPSQRSR) as a control peptide and conjugated them with a fluorescent dye (fluorescein isothiocyanate, FITC) or a radionuclide of ^99m^Tc to evaluate their targeting capacity in 4T1 cancer cell-based experiments. For fluorescence assays, the *N*-termini of both peptides were modified with an additional cysteine to endow them with a sulfhydryl group for FITC attachment to form LyP-1-FITC and ARAL-FITC. For ^99m^Tc radiolabeling, the *N*-termini of these peptides were modified with a hexahistidine tag and radiolabeled with a [^99m^Tc(OH_2_)_3_(CO)_3_]^+^ complex to prepare ^99m^Tc-LyP-1 and ^99m^Tc-ARAL, as this reaction is readily achieved in PBS solution with a high labeling yield and radiochemical purity (RCP) [24]. After evaluating in vitro targeting ability of LyP-1, the localization of LyP-1-FITC in tumor tissues was analyzed by the immunofluorescence method, and the imaging efficacy of ^99m^Tc-LyP-1 was further confirmed in vitro and in a TNBC tumor-bearing mouse model.

## 2. Materials and Methods

### 2.1. Tumor Cell Lines and Tumors

4T1 murine breast carcinoma cells were purchased from American Type Culture Collection (Manassas, VA, USA) and maintained in RPMI-1640 supplemented with 10% fetal bovine serum (FBS, Gibco, Grand Island, NY, USA). To produce breast cancer models, 4-week-old female BALB/c nude mice were purchased from the Shanghai Laboratory Animal Center at the Chinese Academy of Sciences (Shanghai, China) and subcutaneously injected in the left lateral thigh with 1 × 10^6^ tumor cells suspended in 100 *μ*L of PBS. All animal experiments were performed according to protocols established by the Ethics Committee of Shanghai General Hospital.

### 2.2. Synthesis of Fluorescein-Conjugated Peptides

The amino acid sequence of control peptide was ARALPSQRSR which like LyP-1 has the same sum number of basic residues [10] and overall charge [13]. LyP-1 and ARAL were manufactured by Synpeptide Co. Ltd (Shanghai, China). During the synthesis process, the peptides were modified with cysteine at their *N*-termini for FITC labeling [19] or a hexahistidine tag at their *N*-termini for ^99m^Tc radiolabeling.

### 2.3. Synthesis of ^99m^Tc-Conjugated Peptides

The LyP-1 and ARAL peptides were labeled with a [^99m^Tc(OH_2_)_3_(CO)_3_]^+^ complex that was prepared in solution using an in situ CO source. To a septum-sealed glass vial containing 4.5 mg Na_2_[H_3_BCO_2_], 2.85 mg Na_2_B_4_O_7_·10H_2_O, 7.15 mg Na_2_CO_3_, and 8.5 mg Na_2_C_4_H_4_O_6_·2H_2_O under argon atmosphere, 60–120 mCi sodium pertechnetate was added in 1.0 mL physiological saline. The vial was incubated in a boiling water bath for 30 min. After that, the vial was cooled to room temperature, and 175 *μ*L HCl (1.0 M) was added to adjust the pH to 7.0–7.5. Quality control of the [^99m^Tc(OH_2_)_3_(CO)_3_]^+^ complex was performed by instant thin-layer chromatography (ITLC) using silica gel 60 F254 TLC plates (Merck, Darmstadt, Germany) and ITLC-SG glass microfiber chromatography papers (Agilent, Folsom, CA, USA). The mobile phases were 1% HCl in the methanol and citrate buffer (pH = 5.4), respectively. The TLC plates and chromatography papers were analyzed with a thin-layer chromatogram scanner (Bioscan Inc., Tucson, AZ). To radiolabel LyP-1, the [^99m^Tc(OH_2_)_3_(CO)_3_]^+^ complex was added to a sealed vial containing 50 *μ*g LyP-1 in 100 *μ*L phosphate-buffered saline (PBS, pH = 7.4) and the mixture was incubated at 37°C for 1 h to yield ^99m^Tc-LyP-1 [25–27]. The ^99m^Tc-ARAL was prepared using the same experimental conditions. Their radiochemical purities (RCPs) were evaluated by ITLC-SG glass microfiber chromatography papers using citrate buffer (pH = 5.4) as the mobile phase and analyzed by the same TLC scanner. The in vitro radiostabilities were assessed by measuring RCPs at different time intervals as well.

### 2.4. Cytotoxicity Assay

To assess potential cytotoxic effects of LyP-1 or ARAL, cell proliferation assays were conducted with CCK-8 (Dojindo Technologies, Kumamoto, Japan). 4T1 cells (1 × 10^4^ per well) were plated on 96-well plates in the RPMI-1640 medium with 10% FBS. After 24 h, the medium was replaced by 100 *μ*L of fresh containing different concentrations of LyP-1 or ARAL (0.1, 1, 5, 10, 20, or 50 *μ*M) for another 24 h. Then, 10 *μ*L CCK-8 was added for 2 h. Absorbance was measured at 450 nm using a Varioskan Flash multimode microplate reader (Thermo Fisher Scientific, Waltham, MA, USA).

### 2.5. Flow Cytometry

The binding affinity of LyP-1 to 4T1 cells was determined by flow cytometry. In brief, 4T1 cells were seeded into 6-well plates at a density of 2 × 10^5^ cells/well in 2 mL medium and cultured at 37°C for 24 h. Prior to the experiment, cells were washed twice with PBS to remove the remnant medium and then incubated with the serum-free medium containing 10 *μ*M LyP-1-FITC or ARAL-FITC. Cells treated with the drug-free medium containing an equal volume of PBS were used as blank controls. After 2 or 3 h incubation, cells were trypsinized and centrifuged at 1000 rpm for 3 min, washed, and resuspended in 1 mL of PBS. Then, FITC-positive cells were analyzed in the FL1-fluorescence channel using a BD AccuriTM C6 Flow Cytometer (BD Biosciences, Franklin Lakes, NJ, USA). At least 10,000 events were obtained for each sample.

### 2.6. Laser Confocal Microscopy

Uptake of LyP-1 by 4T1 cells was observed with laser confocal microscopy. In brief, 4T1 cells (2 × 10^5^ cells) were seeded into glass-bottom dishes (Cellvis) in 2 mL medium. After 24 h incubation at 37°C and 5% CO_2_, the medium was replaced with 2 mL serum-free medium containing 10 *μ*M FITC-LyP-1 or FITC-ARAL for 3 h. The blank control group was treated with the drug-free medium with an equal volume of PBS. Then, the cells were rinsed three times with PBS, fixed with 4% paraformaldehyde in PBS for 15 min, and stained for nucleic acids with 200 *μ*L of 1 *μ*g/mL DAPI (Beyotime Biotechnology, Haimen, China) for 5 min. After washing with PBS, fluorescent images of cells were analyzed using a Leica SP8 laser confocal microscope (Wetzlar, Germany).

### 2.7. SPECT Imaging In Vitro

For SPECT imaging in vitro, 4T1 cells were cultured under the same conditions described for flow cytometry and incubated with ^99m^Tc-LyP-1 or ^99m^Tc-ARAL at five different radiological doses (25, 50, 100, 200, and 400 *μ*Ci/mL) for 4 h. Then, the cells were washed, trypsinized, and centrifuged at 1000 rpm for 3 min. Next, 4T1 cells were collected in Eppendorf tubes and imaged using a GE Infinia SPECT scanner (GE Healthcare, Chicago, IL, USA) equipped with the Xeleris 2.0 Work Station and a high-energy general purpose collimator for ^99m^Tc. For quantitative analysis, the signal intensities of 4T1 treated with different protocols were evaluated by analyzing the regions of interest.

### 2.8. Immunofluorescence Analysis

To evaluate LyP-1 distribution in tumors, immunofluorescence analyses of frozen tumor sections were performed. First, 500 *μ*L of 1 mM LyP-1-FITC was intravenously injected into a tumor-bearing mouse when the tumor size reached ∼1 cm. After allowing 15 min for circulation [9], three mice were perfused through the heart with 4% paraformaldehyde, and the tumors were harvested. Tissues were cryoprotected in 30% sucrose and frozen in OCT (optimal cutting temperature) embedding medium. Tissues were sectioned at 5 *μ*m and stained with rabbit anti-mouse CD31 or rabbit anti-mouse LYVE-1 (both at 1 : 100; Servicebio, Shanghai, China) at 4°C overnight. Alexa Fluor-594-conjugated goat anti-rabbit antibodies were incubated for 1 h at room temperature. The sections were finally visualized under a fluorescent microscope (Leica).

### 2.9. In Vivo SPECT Imaging

When tumors reached 1.0 cm in diameter, mice were randomly divided into 2 groups (3 per group). After anesthetization with pentobarbital sodium (40 mg/kg), 200 *μ*L solution of ^99m^Tc-LyP-1 or ^99m^Tc-ARAL ([^99m^Tc] = 10 mCi/mL) was injected via the tail vein. SPECT images were acquired at 5 min, 0.5 h, 1 h, 2 h, 4 h, and 6 h postinjection using the GE Infinia SPECT Scanner. To compare the results under the same conditions, the counts of each image were set at 500 K. Next, another 2 groups of 4T1 tumor-bearing mice (3 per group) were injected via the tail vein with ^99m^Tc-LyP-1 or ^99m^Tc-ARAL (200 *μ*L, [^99m^Tc] = 1 mCi/mL) to determine the biodistribution properties. The mice were sacrificed by cervical dislocation at 6 h postinjection, and the major organs including the liver, spleen, kidneys, heart, lung, stomach, intestines, soft tissue, and tumors were collected and weighed. The radioactivity counts of all the samples were measured and expressed as counts per min (cpm) after correction for decay. The results are shown as the percentage of injection dose/gram (%ID/g) of wet tissue and presented as mean ± SD (*n* = 3) for each group.

### 2.10. Statistical Analysis

Data were expressed as means ± standard deviation (SD). Statistical data analysis was analyzed by one-way analysis of variance with *P* < 0.05 as the minimal level of significance. Data were described as follows: ^*∗*^ < 0.05, ^*∗∗*^ < 0.01, and ^*∗∗∗*^ < 0.001, respectively.

## 3. Results and Discussion

### 3.1. Radiolabeling

**Figure 1:**
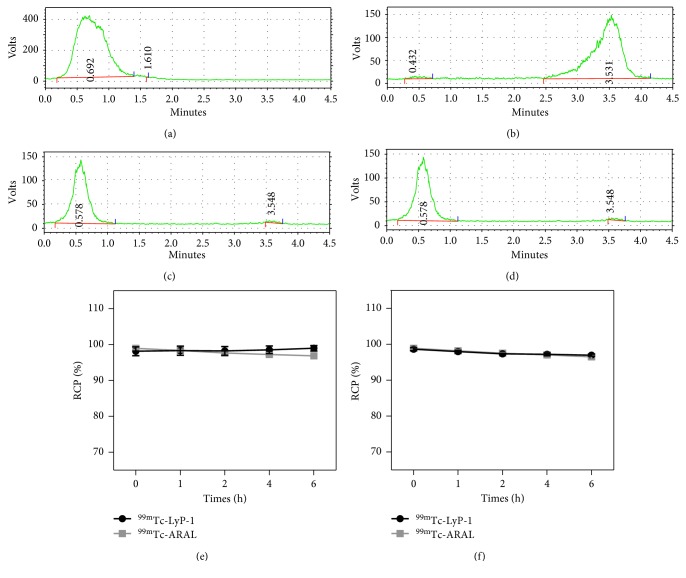
The ITLC results of [^99m^Tc(OH_2_)_3_(CO)_3_]^+^ on TLC plates using the methanol containing 1% HCl as the mobile phase (a) and on ITLC-SG papers using the citrate buffer as the mobile phase (b) the ITLC results of ^99m^Tc-LyP-1 (c) and ^99m^Tc-ARAL (d) on ITLC-SG papers using the citrate buffer as the mobile phase; the radiochemical purities of ^99m^Tc-LyP-1 and ^99m^Tc-ARAL in PBS (e) or FBS (f) at room temperature for 0, 1, 2, 4, and 6 h.

As shown in Figure 1(a), the [^99m^Tc(OH_2_)_3_(CO)_3_]^+^ complex was performed by TLC plates using the methanol containing 1% HCl as the mobile phase. The colloidal ^99m^Tc and ^99m^TcO_4_ have retention factors (Rf) of 0 and 1, respectively, while the [^99m^Tc(OH_2_)_3_(CO)_3_]^+^ is represented by a broad peak with an average Rf = 0–0.5. In Figure 1(b), the [^99m^Tc(OH_2_)_3_(CO)_3_]^+^ complex was evaluated by ITLC-SG papers using the citrate buffer as the mobile phase. The colloidal ^99m^Tc has a Rf of 0, while the [^99m^Tc(OH_2_)_3_(CO)_3_]^+^ and ^99m^TcO_4_ have retention factors (Rf) of 1. In this step, RCP was defined as ^99m^Tc radioactivity associated with [^99m^Tc(OH_2_)_3_(CO)_3_]^+^ as a percentage of total ^99m^Tc radioactivity on the plates and papers. When its RCP was above 95%, the formed [^99m^Tc(OH_2_)_3_(CO)_3_]^+^ was used for the radiolabeling of LyP-1 and ARAL. Similarly, the ^99m^Tc-ARAL and ^99m^Tc-LyP-1 were analyzed by ITLC-SG papers using the citrate buffer mobile phase. As shown in Figures 1(c) and 1(d), both ^99m^Tc-LyP-1 and ^99m^Tc-ARAL had a Rf of 0, and their mean RCPs were >95% without purification after radiolabeling. The ITLC data indicated that both ^99m^Tc-LyP-1 and ^99m^Tc-ARAL have satisfactory stability in PBS or FBS, and their RCPs were >95% even at our longest time point of 6 h at room temperature (Figure 1).

### 3.2. Cytotoxicity

**Figure 2:**
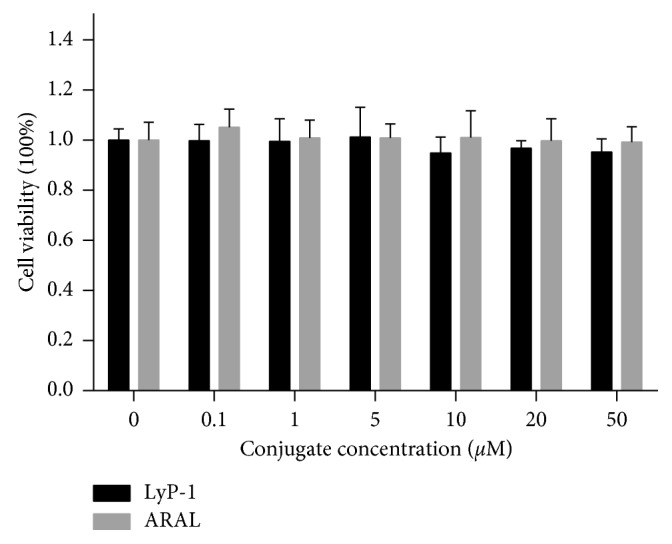
Cytotoxic effects of LyP-1 and ARAL on 4T1 cells at concentrations from 0.1 to 50 *μ*M after incubation for 24 h.

The cytotoxicities of LyP-1 and ARAL were determined by cell counting kit-8 (CCK-8) assays of 4T1 cells after treatment at different concentrations. After 24 h incubation, we found that the viabilities of cells treated with LyP-1 in the concentration range of 0.1–10 *μ*M were all nearly 100%, which was similar to cells treated with PBS or ARAL peptide. Cell viability in a LyP-1 concentration range of 10–50 *μ*M was slightly but not significantly lower than that for 0.1–10 *μ*M (Figure 2). The results show that LyP-1 or ARAL did not exert appreciable cytotoxic effects within the given concentration range. However, LyP-1 was previously reported to be cytotoxic to target cells such as tumor cells and tumor lymphatics [9] and macrophages in atherosclerotic plaques [24]. In MDA-MB-435 tumor cells, LyP-1 can cause a dose dependent increase in cell lysis with a half maximal inhibitory concentration of 66 *μ*M. In mice models, tumor growth can be inhibited by systemic treatment with LyP-1, and tumor lymphatics are more affected than blood vessels. Our results may be due to the shorter inoculation of 24 h and a lower concentration range of 0.1–50 *μ*M. Collectively, our data indicate that LyP-1 could be a safe targeting ligand in conjugation with imaging agents such as fluorescent and radionuclide molecules.

### 3.3. Specificity of LyP-1 to 4T1 Cells In Vitro

**Figure 3:**
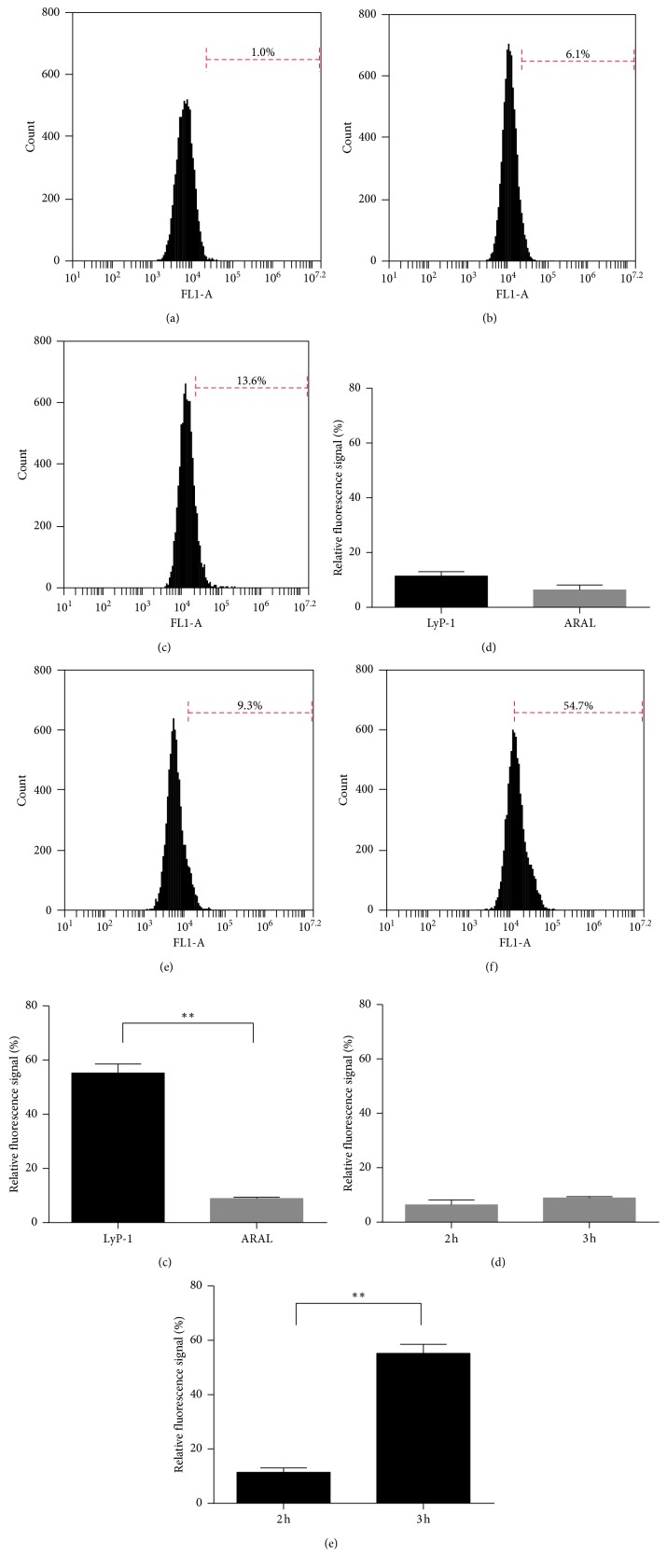
Cellular uptake of LyP-1-FITC and ARAL-FITC by 4T1 cells was examined by flow cytometry. Cells were treated with the drug-free medium (a), ARAL-FITC (b), or LyP-1-FITC (c) at a concentration of 10 *μ*M for 2 h, and the statistical analysis results are shown in (d). Cells treated with 10 *μ*M ARAL-FITC (e) and LyP-1-FITC (f) were analyzed after incubation for 3 h, and the difference was compared in (g). The relative fluorescence signals of cells treated with ARAL-FITC (h) or LyP-1-FITC (i) were compared for 2-3 h incubation, respectively. ^*∗∗*^ < 0.01.

**Figure 4:**
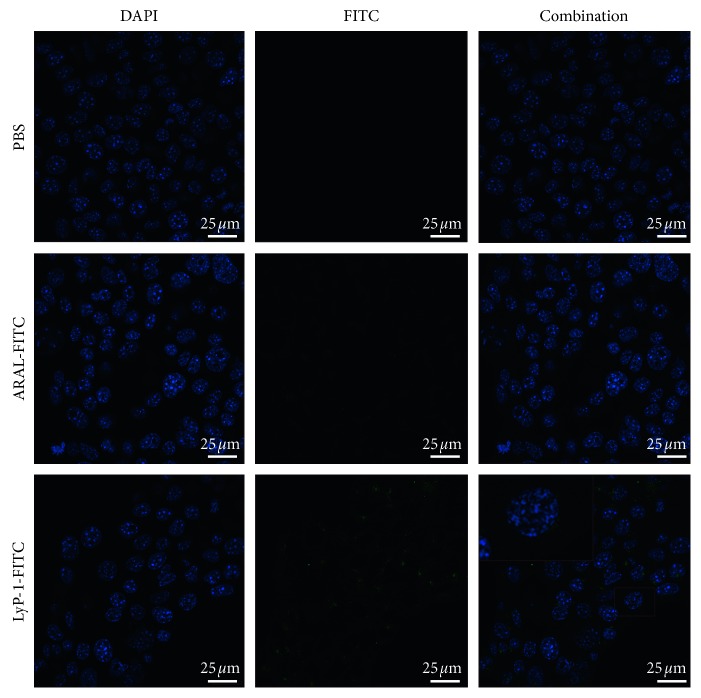
Laser confocal microscopy images of 4T1 cells incubated with ARAL-FITC or LyP-1-FITC at a concentration of 10 *μ*M for 3 h.

Conjugating FITC with LyP-1 enables quantitative detection with flow cytometry and qualitative observation by laser confocal microscopy. We first compared the specific cellular uptakes of LyP-1 and ARAL by flow cytometry. After 2 h incubation, the fluorescence intensity of 4T1 cells treated with LyP-1-FITC was slightly but not significantly higher than those treated with ARAL-FITC (Figures 3(a)–3(d)). When we increased the incubation time to 3 h, the fluorescence intensity of LyP-1-FITC was significantly higher than that of ARAL-FITC (Figures 3(e)–3(g)). Moreover, from 2 to 3 h incubation, the fluorescence intensity in 4T1 cells treated with ARAL-FITC insignificantly increased from 6.1% to 9.3% (Figure 3(h)), whereas the fluorescence intensity in 4T1 cells treated with LyP-1-FITC significantly increased from 13.6% to 54.7% (Figure 3(i)). These results suggest that at least 3 h is needed to obtain better targeting capacity of LyP-1. For laser confocal microscopy, more obvious FITC signal was observed in cells incubated with LyP-1-FITC for 3 h compared to cells treated with ARAL-FITC (Figure 4), further confirming LyP-1 could specifically target 4T1 tumor cells. The majority of LyP-1-FITC fluorescence accumulated in the cytoplasm at 3 h of incubation, which is in accordance with another study [25].

**Figure 5:**
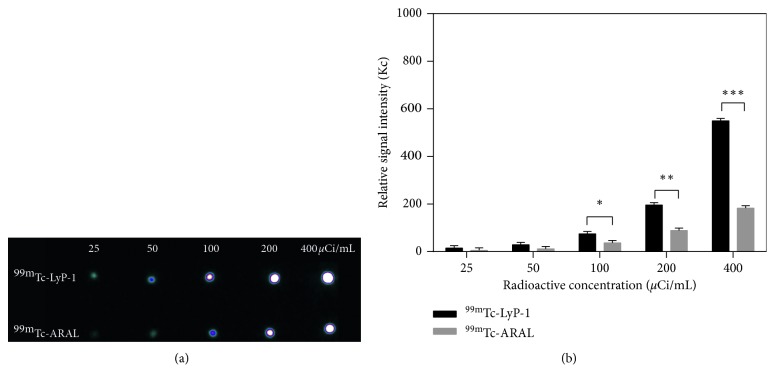
SPECT images of 4T1 cells incubated with ^99m^Tc-LyP-1 and ^99m^Tc-ARAL at different radiodoses for 4 h in vitro SPECT images (a) and their relative SPECT signal intensities (b) of 4T1 cells treated with ^99m^Tc-LyP-1 or ^99m^Tc-ARAL at ^99m^Tc radioactive concentrations of 25, 50, 100, 200, and 400 *μ*Ci/mL, respectively. ^*∗*^ < 0.05, ^*∗∗*^ < 0.01, and ^*∗∗∗*^ < 0.001.

Next, we tested the capacity of LyP-1 in delivering ^99m^Tc to 4T1 cells in vitro. SPECT imaging of 4T1 cells after 4 h incubation is shown in Figure 5. A significantly higher signal intensity was observed in cells treated with ^99m^Tc-LyP-1 than those treated with ^99m^Tc-ARAL at concentration doses of 100, 200, and 400 *μ*Ci/mL instead of 25 and 50 *μ*Ci/mL. At the highest ^99m^Tc concentration of 400 *μ*Ci/mL, the signal intensity of ^99m^Tc-LyP-1 was 3 times higher than that of ^99m^Tc-ARAL. These findings support the flow cytometry and laser confocal microscopy data and confirm the ability of LyP-1 to target 4T1 cells in vitro.

### 3.4. LyP-Recognizes Lymphatics in Tumor In Vivo

**Figure 6:**
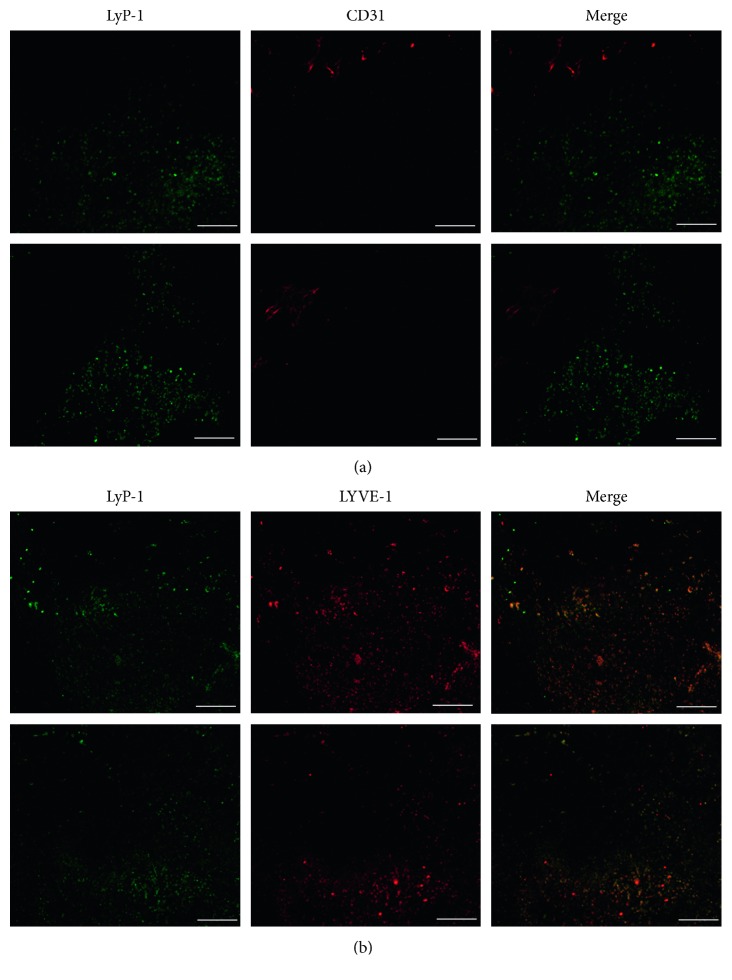
LyP-1 recognizes tumor lymphatics in a 4T1 mouse model. Colocalization of LyP-1-FITC (green) in 4T1 tumor tissue with CD31 (a) (red, a blood vessel marker) and LYVE-1 (b) (red, a lymphatic endothelial marker). Scale bar, 100 *μ*m.

The CendR motif on the sequence of LyP-1 provides LyP-1-based probes with the potential capacity of transvascular transport and parenchymal penetration. Previous studies showed that LyP-1-based probes can reach the tumor vascular region and deep into tumor tissue [13, 23]. In our study, frozen sections of 4T1 tumor xenografts were stained for CD31 (a vascular marker) and LYVE-1 (a lymphatic marker) to investigate the location of LyP-1 in relations to tumor vasculature or lymphatics. The results are shown in Figure 6. LyP-1-FITC distribution was high in close proximity to LYVE-1-positive but CD31-positive regions. These findings corroborate previous reports [23, 25] and imply that LyP-1 can penetrate deep inside tumors and recognize tumor lymphatics.

### 3.5. In Vivo SPECT Imaging of a 4T1 Tumor Mice Model

The ability of LyP-1 to serve as an active targeting peptide for delivering imaging agents to 4T1 cells was verified in vitro. Green fluorescence-labeled cells were much more abundant in samples treated with LyP-1-FITC compared with control peptide-FITC, as assessed by flow cytometry and laser confocal microscopy. In SPECT imaging, ^99m^Tc-labeled LyP-1 also exhibited better targeting ability to 4T1 cells compared to ARAL-FITC. Furthermore, LyP-1 can penetrate tumor blood vessels and spread deeper through the tumor tissue, suggesting that it could effectively deliver imaging agents into tumors. The strong tumor-homing and penetration properties of LyP-1 make it a promising tool for targeted delivery of diagnostic agents to TNBC.

**Figure 7:**
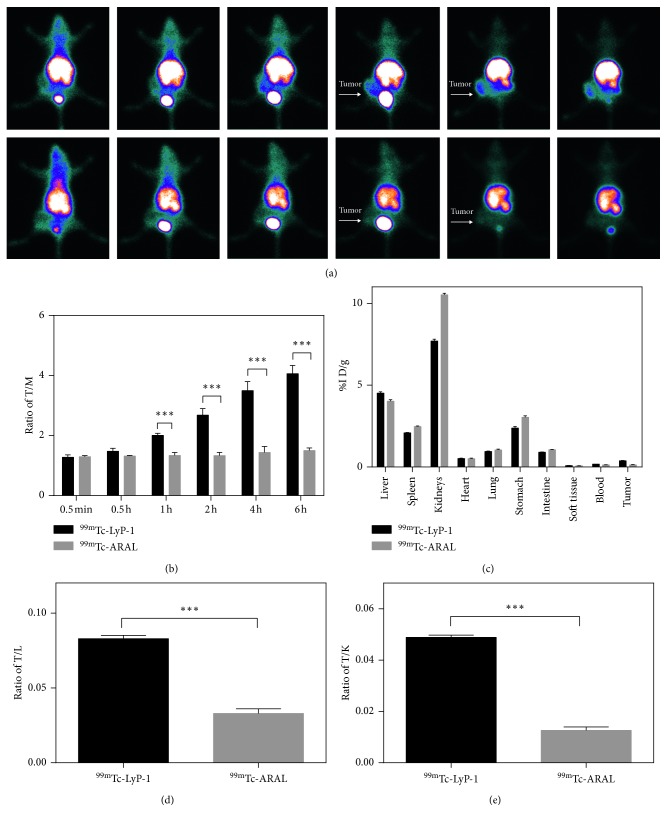
SPECT imaging of ^99m^Tc-LyP-1 in vivo. SPECT images of nude mice bearing 4T1 xenografted tumors at different time points after intravenous injection of ^99m^Tc-LyP-1 (a). The tumor-to-muscle (T/M) ratios of ^99m^Tc-LyP-1 and ^99m^Tc-ARAL were compared at 0.5 min, 0.5 h, 1 h, 2 h, 4 h, and 6 h (b). The relative SPECT signal intensities of ex vivo tumors and major organs (c) at 6 h postinjection of ^99m^Tc-LyP-1 or ^99m^Tc-ARAL. The ratio of tumor/liver (T/L) and tumor/kidney (T/K) of ^99m^Tc-LyP-1 (d) and ^99m^Tc-ARAL (e) was compared at 6 h postinjection. ^*∗∗∗*^ < 0.001.

To investigate whether LyP-1 is effective in vivo, we injected ^99m^Tc-LyP-1 via the tail vein to nude mice bearing 4T1 tumor cells; control animals received ^99m^Tc-ARAL. As shown in Figure 7(a), high relative radioactivity was observed in the abdomen in mice treated with ^99m^Tc-LyP-1 or ^99m^Tc-ARAL at each time point. The bladder SPECT signal intensity increased from 5 min to 2 h, suggesting that both molecules could be cleared by the urinary system. For ^99m^Tc-LyP-1 administration, tumor SPECT signal was first observed at 5 min, was clear at 2 h, and showed the highest signal intensity at 6 h postinjection. In contrast, no tumor SPECT signal intensity changes were observed in mice treated with ^99m^Tc-ARAL over the same time period. The ratios of the tumor-to-muscle (TM) signal at different time points after injection are shown in Figure 7(b). The ratios of TM in mice treated with ^99m^Tc-LyP-1 were 1.3, 1.5, 2.0, 2.7, 3.5, and 4.1 at 5 min, 0.5 h, 1 h, 2 h, 4 h, and 6 h, respectively. Conversely, the ratio of TM in mice treated with ^99m^Tc-ARAL was stable at the range of 1.2–1.5 for the same time points. To further confirm the distinct differences in SPECT signal intensities of tumors treated with ^99m^Tc-LyP-1 or ^99m^Tc-ARAL, a biodistribution experiment was performed at 6 h postinjection (Figure 7(c)). There was no obvious difference in the biodistributions of ^99m^Tc-LyP-1 and ^99m^Tc-ARAL. Similarly, most SPECT signal was in the kidney and liver followed by the stomach and spleen. The lung, intestine, heart, soft tissue, blood, and tumor showed relatively low accumulation of radioactivity signal intensity. Notably, ^99m^Tc-ARAL accumulation in the kidneys was much higher than that of ^99m^Tc-LyP-1, suggesting faster urinary excretion of ^99m^Tc-ARAL. With regard to tumor region, the SPECT signal in mice treated with ^99m^Tc-LyP-1 was significantly higher than that in control mice. In addition, we also quantized the ratio of tumor/liver (T/L) and tumor/kidney (T/K) at 6 h postinjection, and the results further confirmed higher tumor accumulation in tumor of LyP-1 than that of ARAL, as shown in Figures 7(d) and 7(e). Collectively, these data suggest that ^99m^Tc-LyP-1 is a promising imaging agent for TNBC diagnosis.

## 4. Conclusions

In summary, we characterized a novel ^99m^Tc-labeled LyP-1-based imaging probe as a potential SPECT radiopharmaceutical for TNBC diagnosis. The LyP-1 peptide was easily labeled with ^99m^Tc using the [^99m^Tc(OH_2_)_3_(CO)_3_]^+^ complex with high RCP. Moreover, we synthesized a fluorescent molecule LyP-1; its binding ability was tested and verified in vitro, and its penetration property was also confirmed in vivo. Finally, SPECT imaging of mice bearing 4T1 xenografts showed that ^99m^Tc-LyP-1 can be a powerful tool for targeted SPECT imaging of TNBC in vivo.

## Data Availability

The data used to support the findings of this study are available from the corresponding author upon request.
